# Structural analysis of the novel influenza A (H7N9) viral Neuraminidase interactions with current approved neuraminidase inhibitors Oseltamivir, Zanamivir, and Peramivir in the presence of mutation R289K

**DOI:** 10.1186/1471-2105-14-S16-S7

**Published:** 2013-10-22

**Authors:** Chinh Tran-To Su, Xuchang Ouyang, Jie Zheng, Chee-Keong Kwoh

**Affiliations:** 1 Bioinformatics Research Center, School of Computer Engineering, Nanyang Technological University, Singapore 639798; 2 Genome Institute of Singapore, Agency of Science, Technology, and Research (A*STAR), Biopolis, Singapore 138672

## Abstract

**Background:**

Since late March 2013, there has been another global health concern with a sudden wave of flu infections by a novel strain of avian influenza A (H7N9) virus in China. To-date, there have been more than 100 infections with 23 deaths. It is more worrying as this viral strain has never been detected in humans and only been found to be of low-pathogenicity. Currently, there are 3 effective neuraminidase inhibitors for this H7N9 virus strain, i.e. oseltamivir, zanamivir, and peramivir. These drugs have been used for treatment of the H7N9 influenza in China. However, how these inhibitors work and affect the binding cavity of the novel H7N9 neuraminidase in the presence of potential mutations has not been disclosed. In our study, we investigate steric effects and subsequently show the conformational restraints of the inhibitor-binding site of the non-mutated and mutated H7N9 neuraminidase structures to different drug compounds.

**Results:**

Combination of molecular docking and Molecular Dynamics simulation reveal that zanamivir forms more favorable and stable complex than oseltamivir and peramivir when binding to the active site of the H7N9 neuraminidase. And it is likely that the novel influenza A (H7N9) virus adopts a higher probability to acquire resistance to peramivir than the other two inhibitors. Conformational changes induced by the mutation R289K causes loss of number of hydrogen bonds between the inhibitors and the H7N9 viral neuraminidase in 2 out of 3 complexes. In addition, our results of binding-affinity relationships of the 3 inhibitors with the viral neuraminidase proteins of previous pandemics (H1N1, H5N1) and the current novel H7N9 reflected the extent of binding effectiveness of the 3 inhibitors to the novel H7N9 neuraminidase.

**Conclusions:**

The results are novel and specific for the A/Hangzhou/1/2013(H7N9) influenza strain. Furthermore, the protocol could be useful for further drug-binding analysis and prediction of future viral mutations to which the virus evolves through adaptation and acquires resistance to the current available drugs.

## Background

There has been another global health concern since the last few months by the emergence of a novel strain of avian influenza A (H7N9) virus, which has never been detected in humans [1,2]. The virus has infected more than 100 with 23 deaths as of April 16, 2013 [3]. According to World Health Organization (WHO), this avian influenza A (H7N9) strain is considered to be one of the most lethal influenza viruses [4] because reported infections occur sporadically, and asymptomatically (i.e. one patient case found in Beijing, China) [2]. This novel low-pathogenic H7N9 strain does not cause disease symptoms in animals; hence it easily escapes detection from animal reservoir and has higher probability to transmit than the previous highly pathogenic H5N1 strain, which killed hundreds worldwide [5,6]. Even though there has been no epidemiological evidence of direct transmission between humans, signs of viral adaption to humans via its mutations have been detected [7,8]. Therefore, it could be just a matter of time before the new strain of virus can pose a potential human pandemic.

Genetic analysis have shown that H7N9 virus could acquire through adaptation the ability to infect mammals (especially humans) better than other avian influenza strains [1,9] via critical mutations [5,10]. The novel H7N9 virus is known to be susceptible to neuraminidase inhibitors oseltamivir and zanamivir. Recently, another antiviral drug peramivir has been approved for H7N9 influenza treatment in China. These drug compounds inhibit enzymatic activity of the viral neuraminidase, which has a role in the final step of sialic acid cleavage that helps release the virus from the infected cells [11].

Gene mutations that cause viral resistance to most of the drugs have raised significant concern because they may trigger potential pandemics. Typical well-established mutation His274Tyr (N2 numbering) within the neuraminidase (NA) has been known to confer a very high level of resistance to oseltamivir without compromising viral fitness in the highly pathogenic influenza viruses (H5N1 and H1N1) of both the previous pandemics [12-16]. Russell *et al. *found that there are substantial conformational differences adjacent to the binding sites between group-1 (N1, e.g. H5N1, H1N1) and group-2 (N9, e.g. H7N9) neuraminidases [15], causing this H274Y mutation against oseltamivir to have little effect on N9 neuraminidase compared to the other NA group [15,16]. Instead, the novel H7N9 has acquired other gene mutations to adapt itself more "human-like" [5,10]. In fact, all H7N9 specimens in China show a deletion of five residues (position 69-73) in the viral NA stalk compared to the avian-origin influenza A (H7N9) [17], and it was once found to increase virulence in mice [18]. So far, a gene mutation for Arg292Lys (R292K, N2 numbering) found in the first case of H7N9 (/Shanghai/1/2013) in China causes reduced drug susceptibility to oseltamivir and zanamivir [17,19]. Interaction mechanism of the substituted residue Lys^292 ^in the binding sites of some viral N1 and N9 neuraminidases were investigated [15,20]. However, how this R292K (R289K in H7N9 numbering) mutation affects the inhibitor-binding site of the novel avian influenza A (H7N9) virus has not yet been understood. Therefore, our work aims to provide an insight into the conformational changes of the novel H7N9 neuraminidase binding site in the presence of the mutation. With this, we hope to understand how these steric changes affect bindings of the three inhibitors oseltamivir, zanamivir, and peramivir.

## Results and discussion

### Mutation R289K causes different conformational changes in the structure of the H7N9 Neuraminidase when it interacts with oseltamivir, zanamivir, and peramivir

We used 3 current approved neuraminidase inhibitors (i.e. oseltamivir, zanamivir, and peramivir), which are applied in the treatment of the influenza A (H7N9), to dock independently as ligand to the non-mutated structure of the H7N9 neuraminidase (NA) using Autodock 4.2 [21]. Subsequently, we performed molecular dynamics (MD) simulation using AMBER 11 [22] for each complex of NA/inhibitor to obtain optimal bound conformations (see Methods). As observed in our docking results, all the inhibitors bind to active site residues of the H7N9 NA (Table 1). Among the 3 NA inhibitors, zanamivir interacts more favorably with the H7N9 NA than oseltamivir and peramivir since the complex of NA/zanamivir obtains lower binding free energy (-54.73 kcal/mol) than complexes of the other two compounds (-49.49 kcal/mol and -39.02 kcal/mol respectively) (Table 2).

**Table 1:** Interactions between three NA inhibitors and the H7N9 NA binding site residues

NA Inhibitor	Non-mutated NA	R289K-mutated NA	#H-bond*
Oseltamivir	Arg^367^, Glu^422^	Arg^367^, Glu^422^	2/2
Zanamivir	Arg^152^, Glu^422^	Glu^422^, **Lys^289^**	4/3
Peramivir	Glu^274^	Glu^422^	3/2

*numbers of hydrogen bond between the inhibitor and NA: before/after mutation occurs

**Table 2:** Binding free energy *ΔΔG *(kcal/mol) of the H7N9 NA/inhibitors complexes

Complex	Non-mutated NA	R289K-mutated NA
NA/oseltamivir	-49.49	-37.46
NA/zanamivir	-54.73	-47.24
NA/peramivir	-39.02	-37.26

To detect how the conformation of the H7N9 NA changes due to the mutation R289K and in the presence of the inhibitors, we substituted the residue Lys (K) for Arg (R) at the position 289 (292 in N2 numbering) using DeepView v4.1 [23]. Minimization and 20-nanosecond MD simulation were performed for the new mutated NA/inhibitor complexes to reach the equilibrium states. Our results indicate that the mutation R289K may result in reducing the drug susceptibility of the viral strain A/Hangzhou/1/2013 (H7N9) to oseltamivir, zanamivir, and peramivir. In fact, except for the case of NA/oseltamivir complex, losses of hydrogen bonds between the inhibitors and the enzyme active sites due to R289K-induced conformational changes of the NA structure are observed (Table 1).

**Figure 1:**
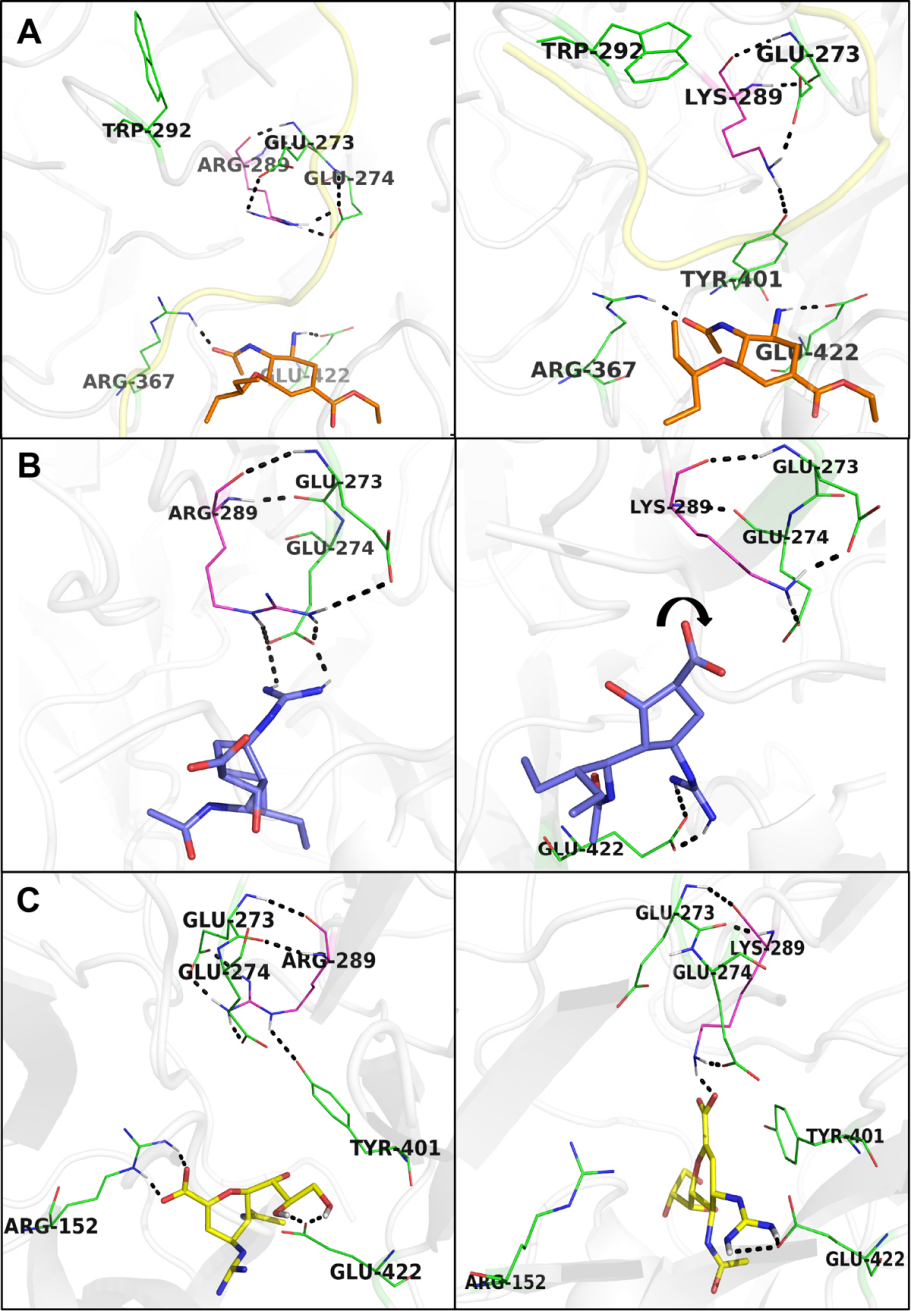
**Observed conformational changes in the inhibitor-binding site of the non-mutated (left) and mutated (right) complexes of NA/inhibitors**. Neuraminidase inhibitors are presented in colored sticks (oseltamivir: orange, peramivir: blue, zanamivir: yellow), other involved NA residues are presented in green line, except for residues Arg and Lys (mutation R289K) in magenta color. The grey cartoon-presented background is the H7N9 NA structure. All the interactions are shown in black dotted line. Oxygen atoms are in red, nitrogens are in blue, and hydrogens are in white. For simplicity, only hydrogens involved in the interactions are shown. (A) Occupation of the bulky residue Trp^292 ^in the empty space created by a substituted smaller residue Lys^289 ^(R289K) and the change of N-terminal (yellow) conformation influenced contacts between oseltamivir and the H7N9 NA binding site. (B) Conformation of peramivir twists (small arrow) towards the empty space, causing it to interact with Glu^422 ^instead of Glu^274^. (C) Conformational change of Tyr^401 ^induces interaction between zanamivir and the substituted residue Lys^289^. However, this interaction also causes one hydrogen bond loss in total between zanamivir and the H7N9 NA.

Structural comparisons between the non-mutated and the mutated NA/inhibitor complexes reveal steric variations of the active site residues and the inhibitor-bound orientations to explain various conformational adaptions of the NA binding pocket in the presence of different inhibitors. In the case of oseltamivir, mutation R289K does not cause any hydrogen bond loss; however, according to our hydrogen bond analysis (see Additional file 1), occurrence of the 2 hydrogen bonds (i.e. between the NA residues Arg^367^, Glu^422 ^and the oseltamivir) decreases during the 20ns MD simulation process. It suggests that oseltamivir may be losing contacts with the H7N9 NA during the viral adaption. While the residue Arg^289 ^interacts with Glu^274 ^and Glu^273^, the substituted smaller residue Lys^289 ^interacts with Tyr^401 ^and Glu^273^. This creates extra empty space, which could be large enough for an interference of the bulky residue Trp^292^; subsequently induces contacts among other neighboring residues Trp^292^, Asn^342^, and Thr^11^. These interactions change the conformations of the enzyme N-terminus and surrounding residues, i.e. Gln^5^, Thr^144^, and Ile^145^; consequently result in losing contacts with oseltamivir (Figure 1A).

Similar to the case of oseltamivir, substitution of a smaller residue Lys^289 ^increases the empty space among residues surrounding the binding sites of the NA/peramivir complex. In this mutated complex, peramivir adapts itself to the changes of the H7N9 NA conformation by making a clockwise twist towards the empty space to interact with Glu^422 ^instead (while it interacts with Glu^274 ^in the non-mutated complex). However, this slight change results in one hydrogen bond loss between peramivir and the viral NA.

It is observed that in the non-mutated NA/peramivir complex, residue Arg^289 ^interacts indirectly with peramivir via two other residues Glu^273 ^and Glu^274 ^serving as intermediate bridges. In contrast, those interaction-mediated hydrogen bonds are not present in the mutated complex. When the mutation R289K occurs, the residues Glu^273 ^and Glu^274 ^change their conformations and interact only with Lys^289 ^without perturbing peramivir (Figure 1B), causing a decrease in the binding affinity with the inhibitor.

Our results show that interaction of the H7N9 NA and zanamivir is the most surprising among the three NA/inhibitors complexes because the mutation R289K produces a direct contact between zanamivir and the substituted Lys^289^. In the non-mutated NA/zanamivir complex, besides two polar contacts with Glu^273 ^and Glu^274 ^(as similar in the non-mutated complex of oseltamivir or peramivir), the residue Arg^289 ^interacts with a third residue Tyr^401^. The bulky residue Tyr^401 ^with its planar aromatic ring serves as an obstruction preventing the contact between zanamivir and Arg^289 ^(Figure 1C). When the smaller residue Lys substitutes Arg, subsequently causing the conformational change of Tyr^401^, this obstruction seems to be alleviated. As a result, zanamivir is pulled upwards and interacts with the substituted Lys^289^. However, changes of zanamivir conformation make it further distant to the active site residue Arg^152 ^(11.9Å) and lose their native interaction. This causes one hydrogen bond loss between zanamivir and the NA protein.

In general, the mutation R289K induced conformational changes of the H7N9 NA inhibitor-binding pocket. Neighboring active site residues, i.e. Glu^273^, Glu^274^, and Tyr^401 ^are involved in those steric alterations to compensate for the decrease of either hydrophilicity or size of the substituted Lys^289^. The mutation results in the loss of number of hydrogen bonds between the inhibitor and the H7N9 NA in 2 out of 3 cases of mutated complexes, i.e. NA/zanamivir, and NA/peramivir. It is too early to claim that the interaction observed in this study occur to all NA proteins of the novel avian influenza A (H7N9) virus, and these results will require further analysis since they are subject to specific docking parameters and MD simulation time (i.e. 20 ns in this study) to achieve the optimal and stable conformations. Also, the results are interpreted for the strain A/Hangzhou/1/2013 (H7N9) from which the mutation has not yet been reported.

### Binding-affinity relationships of oseltamivir, zanamivir, and peramivir with viral neuraminidase proteins of previous pandemic (H1N1, H5N1) and the current novel H7N9

To further investigate the binding effectiveness of the 3 NA inhibitors to the novel H7N9 neuraminidase compared to the previous pandemic influenza A viruses H5N1 and H1N1, we performed molecular docking experiments using AutoDock 4.2 for these inhibitors (as ligand) with several available structures of H5N1 and H1N1 neuraminidases. Their binding affinities (kcal/mol) are tabulated in Table 3.

**Table 3:** Predicted binding affinity of three NA inhibitors with the novel H7N9, H5N1, and H1N1 neuraminidases

NA inhibitor	Binding affinity (kcal/mol)						
	
	Novel H7N9	H5N1 [PDB id]			H1N1 [PDB id]		
		
		[2HTU]	[2HU0]	[2HU4]	[3TI6]	[3B7E]	[3TI5]
oseltamivir	-7.49	-8.65	-8.30	-8.33 (2.26)*	-8.18 (1.82)*	-8.28	-8.16
peramivir	-7.20	-8.53 (1.6)*	-7.35	-7.94	-7.78	-7.81	-7.96
zanamivir	-6.33	-7.08	-6.94	-6.71	-6.92	-7.27 (0.69)*	-7.32 (0.92)*

* Root Mean Square deviation (RMSD) of the docked complex to the native complex structures

Russell *et al. *shows that difference in oseltamivir-binding affinity between group-1 (N1) and group-2 (N9) neuraminidases is not very large [15]. Our results are in accordance with their observation in the case of oseltamivir. Compared to the other two H5N1 and H1N1 neuraminidases in our results, the novel H7N9 NA interacts with all the three inhibitors with higher binding energy. This could suggest that even without the emergence of any mutations, the H7N9 viral susceptibility to these drugs is lower than the other influenza viruses. Hence, it might be easier for this novel H7N9 strain to acquire resistance to these drugs.

In addition, based on our study of binding free energy between the H7N9 NA and the 3 inhibitors, it is likely that the novel influenza A (H7N9) virus adopts a higher probability to acquire resistance to peramivir than the other two inhibitors. Our MD simulation results also indicate that at the equilibrium state the interaction between the H7N9 NA and peramivir is less favorable than those in complexes of NA/oseltamivir and of NA/zanamivir since the NA/peramivir complex obtains the highest binding free energy among the 3 complex associations both before and after (-39.02/-37.26 kcal/mol) the mutation R289K occurs (i.e. NA/oseltamivir: -49.49/-37.46 kcal/mol and NA/zanamivir: -54.73/-47.24 kcal/mol) as shown in Table 2. In future drug design, we expect stronger binding inhibitors will overcome the energy barriers caused by potential mutations of the novel H7N9 influenza A virus.

## Conclusions

Combination of molecular docking and molecular dynamics simulation demonstrated differential inhibitor-bindings of the non-mutated and mutated novel H7N9 influenza viral neuraminidase. Mutation R289K-induced conformational changes that cause the loss of hydrogen bond chains between the inhibitors and the viral neuraminidase suggest potential adaptions of the virus itself for future drug-resistance. While the results are novel for the A/Hangzhou/1/2013(H7N9) strain, further studies and analysis of conformational changes in population level of other strains still needs to be conducted and experimentally verified.

## Methods

### Homology modeling of the novel avian influenza A (H7N9) viral neuraminidase

We used the neuraminidase (NA) sequence of A/Hangzhou/1/2013(H7N9) strain from NCBI [24] [Genbank:AGI60300.1] as our protein target. Examining protein sequences of available N9 neuraminidase structures in Protein Data Bank [25], we observed that the novel H7N9 neuraminidase sequence contains an extra 77 residues at its N-terminus. Therefore, to obtain full-length 3-dimensional structure of this novel protein, we applied homology modeling method using I-TASSER [26] for the selected NA sequence (see Additional file 2).

It was shown that except for the 77-residue region at the N-terminus, the rest of the target NA H7N9 sequence (residues 78-465) is highly similar to all the threading templates (95%). To model the 77-residue N-terminus without any available templates, the I-TASSER server applied replica-exchange Monte Carlo simulation method to simulate the folding process of this region, which contains 2 helices and coils. We observed that the N-terminal 77-residue region was not involved in the inhibitor-binding ability of the H7N9 NA protein; therefore we accepted the best model from I-TASSER and subsequently applied molecular dynamics (MD) simulation method (see below) for the whole model to improve its quality and obtain the optimal conformation.

RMSD calculation of this optimal H7N9 NA structure and two other wild-type N9 neuraminidases [PDB:2QWK] (RMSD = 2.27Å) and [PDB:7NN9] (RMSD = 2.23Å) proved it as a reliable modeled structure. Hence, we used this optimal conformation of H7N9 NA for further analysis.

We performed binding site prediction using SiteHound [27] for this H7N9 NA structure and cross-validated the results with other influenza viral NAs [15]. The predicted binding site contains residues Thr^144^, Ile^145^, Asp^147^, Arg^152^, Glu^274^, Arg^367^, Trp^398^, Ser^399^, Tyr^401^, Glu^422^, and Ile^424^.

### Obtain optimal complexes of non-mutated and mutated H7N9 neuraminidase (NA) with current NA inhibitors

We obtained structures of three NA inhibitors oseltamivir [CID:65028], zanamivir [CID:60855], and peramivir [CID:151164] from PubChem. For all these NA inhibitors, we applied molecular docking method using AutoDock 4.2 [21] to obtain the NA/inhibitor complexes. The inhibitor was docked as ligand to the active site of the H7N9 NA. We used the grid spacing of 0.2 for better accuracy after various docking tests (data not shown). All available rotatable bonds of each ligand (oseltamivir: 11 bonds, zanamivir: 10 bonds, peramivir: 10 bonds) were activated to allow full flexibility of the ligand. For each complex, 100 independent docking experiments were performed, and the top rank-1 conformation was selected for further investigation.

To improve bound conformations of semi-rigid docking from AutoDock 4.2, we conducted molecular dynamics simulations using AMBER 11 force field *ff99SB *[28]. A 3-stage dynamic simulation of minimization, heating, and equilibration under periodic boundary condition was performed for the NA/inhibitor docked complexes. During this process, explicit solvent model was applied. In the first stage, we used a weak positional restraint by a 500kcal/molÅ^2 ^force constant for the whole NA/inhibitor complex of the first 1,000 steps of minimization to hold it fixed while positions of solvent and sodium ions were initially minimized. In the next 2,500 steps of minimization, we removed this restraint. Constant volume was set during the minimization stage. In the next stage of 20ps heating, we put the restraint on the system again, but with only 10kcal/molÅ^2 ^to avoid wild fluctuations within the structure. We heat up the system from 0K to 300K, and in order to control the temperature we applied Langevin temperature equilibration scheme. Then, we performed a 20 ns equilibration stage without the restraints for the whole complex at constant pressure of 1 atm and at 300K. For both the heating and equilibration stages, we applied SHAKE to constrain bonds that involves hydrogen. Finally, we extracted the lowest energy complexes obtained from the 20 ns equilibration above and used them as final bound conformations of the docked NA/inhibitor complexes for further analysis.

### Binding free energy calculation for associations of H7N9 NA protein and NA inhibitors

We used Molecular Mechanics - Generalized Born Surface Area (MM-GBSA) method implemented in AMBER 11 to calculate the binding free energy for the complex of NA and NA-inhibitors. Final estimated binding free energy (*ΔΔG *kcal/mol) is calculated as ΔΔG_binding _= ΔG_complex _- (ΔG_receptor _+ ΔG_ligand_).

For effective computational cost, we only performed the calculation of interaction energy and solvation free energy of the complex, receptor, and ligand, but ignored the entropy contribution to these bindings. Therefore, the free energy (*ΔG*) of each component (receptor, ligand, or complex) consists of various potential terms, i.e. van der Waals, electrostatic energy calculated by molecular mechanics force field, electrostatic contribution and nonpolar contribution to the solvation free energy.

## List of abbreviations used

NA: neuraminidase; MD: molecular dynamics; ns: nanosecond; ps: picosecond; RMSD: root mean square deviation

## Competing interests

The authors declare that they have no competing interests.

## Authors' contributions

CTTS and XO carried out the experiments. CTTS, XO, JZ, and CKK wrote the manuscript. All authors read and approved the final manuscript.

## Supplementary Material

Additional file 1 **Results of hydrogen bond analysis of the 3 NA/inhibitor complexes for the whole 20ns MD simulation**. Click here for file

Additional file 2 **Homology modeling study of the H7N9 NA structure from I-TASSER and MD simulation**. Click here for file

## Declarations

Publication of this work is supported by the Singapore MOE AcRF Tier 2 grant ARC9/10 (MOE2010-T2-1-056), and MOE AcRF Tier 1 grant (RG32/11).

This article has been published as part of *BMC Bioinformatics *Volume 14 Supplement 16, 2013: Twelfth International Conference on Bioinformatics (InCoB2013): Bioinformatics. The full contents of the supplement are available online at http://www.biomedcentral.com/bmcbioinformatics/supplements/14/S16.
