# Supplementary material for: Structural analysis of the novel influenza A (H7N9) viral Neuraminidase interactions with current approved neuraminidase inhibitors Oseltamivir, Zanamivir, and Peramivir in the presence of mutation R289K
Source: BMC Bioinformatics. 2013 Oct 22;14(Suppl 16):S7. doi: 10.1186/1471-2105-14-S16-S7 (PMC3853198; doi:10.1186/1471-2105-14-S16-S7)
Supplement: Additional file 1 — Results of hydrogen bond analysis of the 3 NA/inhibitor complexes for the whole 20ns MD simulation. [file 1471-2105-14-S16-S7-S1.PDF]

Additional-file_1: Results of hydrogen bond analysis of the 3 NA/inhibitor complexes for the whole 20ns MD simulation

NA complex	Donor	Acceptor_H	Acceptor	Occupancy* (%)	Distance (Å)	Angle (^{o})
Oseltamivir (OSE)						
Non-mutated	Glu^{422}@OE2	OSE@HN3	OSE@N1	99.92	2.71	16.45
	OSE@O3	Arg^{367}@HH11	Arg^{367}@NH1	90.95	2.80	20.47
Mutated	Glu^{422}@OE2	OSE@HN3	OSE@N1	99.33	2.73	19.82
	OSE@O3	Arg^{367}@HH11	Arg^{367}@NH1	89.95	2.80	19.29
Zanamivir (ZAN)						
Non-mutated	Glu^{422}@OE2	ZAN@HO	ZAN@O2	97.78	2.67	13.83
	Glu^{422}@OE2	ZAN@HO1	ZAN@O3	96.38	2.67	18.82
	ZAN@O6	Arg^{152}@HH21	Arg^{152}@NH2	88.32	2.80	23.11
	ZAN@O5	Arg^{152}@HE	Arg^{152}@NE	59.55	2.87	23.04
Mutated	ZAN@O5	Lys^{289}@HZ3	Lys^{289}@NZ	47.98	2.77	20.97
	Glu^{422}@OE2	ZAN@HN2	ZAN@N2	34.62	2.83	22.08
	Glu^{422}@OE2	ZAN@HN3	ZAN@N2	23.42	2.84	21.24
Peramivir (PER)						
Non-mutated	Glu^{274}@OE1	PER@HN4	PER@N3	62.15	2.80	27.84
	Glu^{274}@OE2	PER@HN2	PER@N2	36.65	2.83	27.8
	Glu^{274}@OE2	PER@HN3	PER@N2	13.57	2.85	27.43
Mutated	Glu^{422}@OE2	PER@HN4	PER@N3	43.55	2.79	25.9
	Glu^{422}@OE1	PER@HN3	PER@N2	39.55	2.85	26.89

*percentage of hydrogen bond occurrence during the process of 20ns MD simulation
