# Supplementary material for: Structural analysis of the novel influenza A (H7N9) viral Neuraminidase interactions with current approved neuraminidase inhibitors Oseltamivir, Zanamivir, and Peramivir in the presence of mutation R289K
Source: BMC Bioinformatics. 2013 Oct 22;14(Suppl 16):S7. doi: 10.1186/1471-2105-14-S16-S7 (PMC3853198; doi:10.1186/1471-2105-14-S16-S7)
Supplement: Additional file 2 — Homology modeling study of the H7N9 NA structure from I-TASSER and MD simulation. [file 1471-2105-14-S16-S7-S2.PDF]

Additional-file_2: Homology modeling study of the H7N9 NA structure from I-TASSER and MD simulation

The neuraminidase (NA) sequence of A/Hangzhou/1/2013(H7N9) strain from NCBI [1] [Genbank:AGI60300.1] was used as input for the I-TASSER server [2, 3] to obtain the homologous 3D-structures.

For preliminary sequence alignments, I-TASSER applied its local meta-threading approach (LOMETS [4]) to generate a list of templates for modeling (e.g. 1f8e:A, 1f8d:A, 1nca:N, 1ncc:N, 1nmb:N shown in Figure S1). It was shown that except for the 77-residue region at the N-terminus, the target NA sequence is highly similar to all the threading templates (95%) (Table S1).

Table S1: Result of the selected threading templates and their Z-scores by I-TASSER

#	Pdb/chain	Identity1	Identity2	Coverage	Normalized Z-score
1	1f8eA	0.95	0.79	0.83	9.36
2	1nmbN	0.95	0.79	0.83	7.25
3	1ncaN	0.95	0.79	0.83	7.2
4	1nccN	0.95	0.79	0.84	5.16
5	1f8dA	0.95	0.79	0.83	4.91

Identity1 is the percentage sequence identity of the templates in the threading aligned region with the query sequence.
Identity2 is the percentage sequence identity of the whole template chains with query sequence.
Coverage represents the coverage of the threading alignment and is equal to the number of aligned residues divided by the length of query protein.
Normalized Z-score is the normalized Z-score of the threading alignments. Alignment with a normalized Z-score >1 means a good alignment and vice versa.

In addition to sequence alignment, I-TASSER performs structural alignment to determine the structures with high structural similarity to the target (Table S2).

Table S2: Templates with high structural similarity to the target structure (identified by TM-align)

Rank	Pdb/chain	TM-score	RMSD	Structural Identity	Coverage
1	1ncaN	0.834	0.47	0.946	0.837
2	1v0zD	0.828	0.78	0.691	0.834
3	3tiaA	0.811	1.19	0.494	0.828
4	3ti8A	0.810	1.37	0.411	0.832
5	2aepA	0.810	1.22	0.478	0.828

6	3nssA	0.805	1.33	0.484	0.826
7	2htwA	0.805	1.38	0.423	0.828
8	3b7eB	0.804	1.33	0.480	0.824
9	2htrA	0.803	1.39	0.427	0.826
10	1a4gB	0.776	2.03	0.297	0.819

Coverage represents the coverage of the alignment by TM-align and is equal to the number of structurally aligned residues divided by length of the query protein.

We observed that the N-terminal 77-residue region (Figure S1) was not involved in the inhibitor-binding region of the H7N9 NA protein (i.e. around predicted binding residues Thr^{144}, Ile^{145}, Asp^{147}, Arg^{152}, Glu^{274}, Arg^{367}, Trp^{398}, Ser^{399}, Tyr^{401}, Glu^{422}, and Ile^{424}); therefore we accepted the best model from I-TASSER and subsequently applied molecular dynamics (MD) simulation (process of minimization, 20ps heating, 20ns equilibration) for the whole model to improve its quality and obtain the optimal conformation.

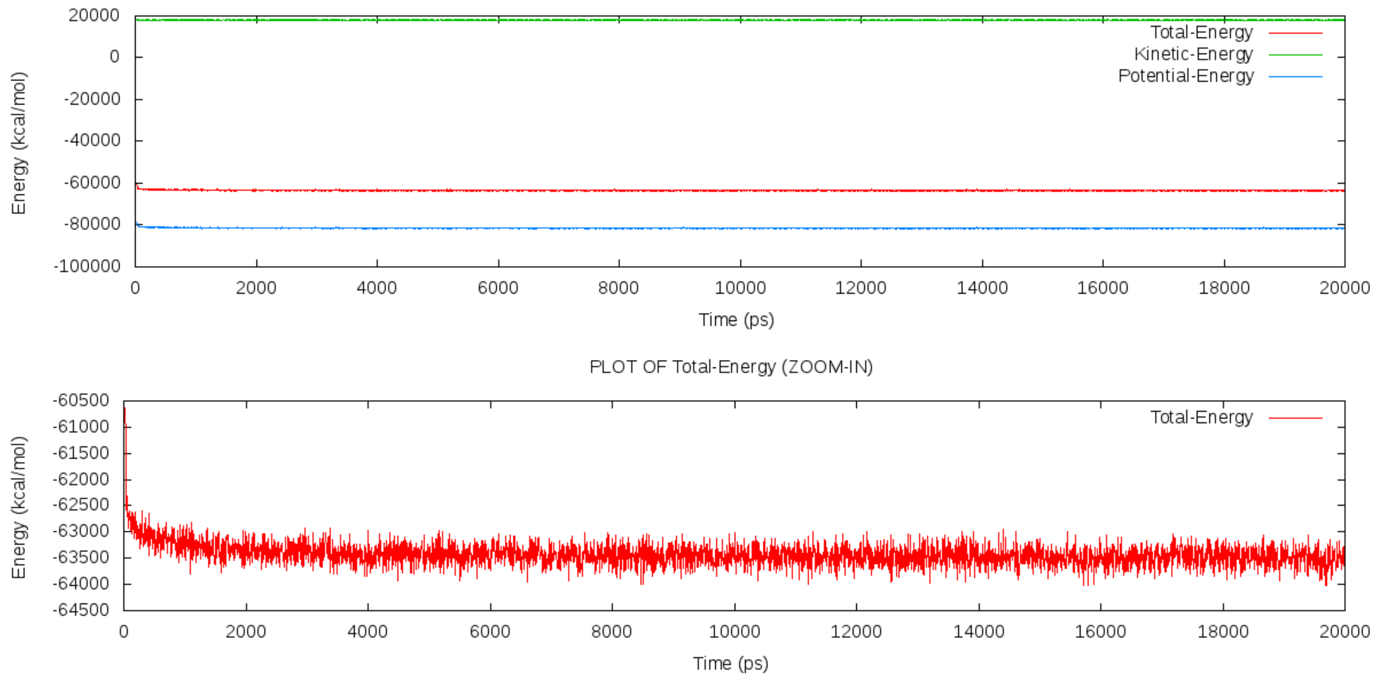

Figure S2: Energy plot during 20ns equilibrium states of the optimal H7N9 NA conformation

The Figure S2 shows a plot of 3 types of energy (i.e. total energy, kinetic energy, and potential energy) during the 20ns equilibration phage in the upper panel, and the zoomed-in total energy plot in the bottom panel. It is indicated that both the kinetic and potential

energies are stable. As we expected, the total energy shows an initial decrease at around the first 1 ns caused by the transition from the previous heating phage. It then stabilizes during the rest of the equilibration, inferring that the NA structure has reached its more stable conformation. From these results, we extracted the conformation that obtained lowest total energy (E ~= -64007 kcal/mol) and used it for further analysis as we assumed that this lowest-energy conformation was the most optimal structure.

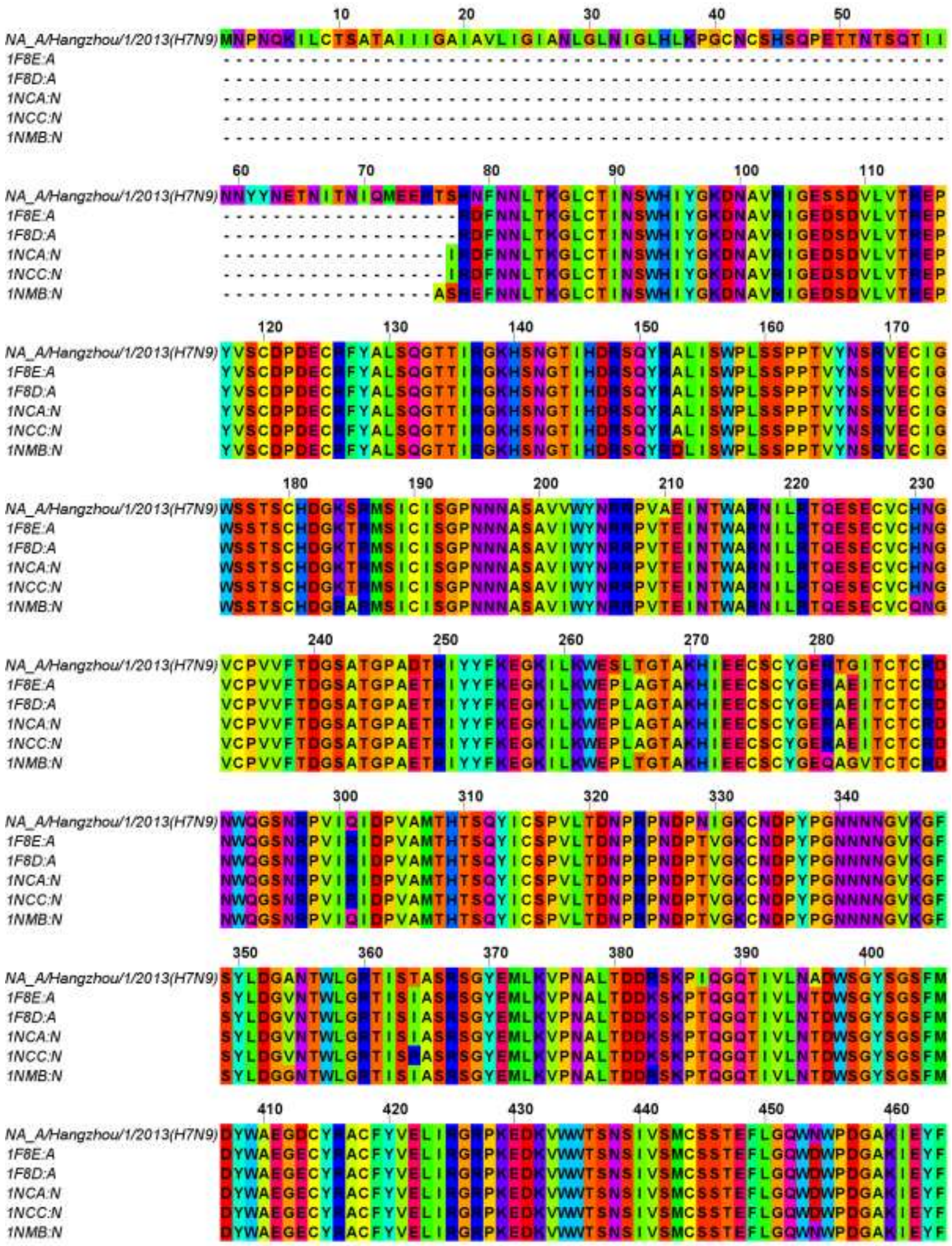

Figure S1: Sequence alignment of the A/Hangzhou/1/2013(H7N9) NA target sequence and selected threading templates

References

1. **National Center for Biotechnology Information** [www.ncbi.nlm.nih.gov]
2. Zhang Y: **I-TASSER server for protein 3D structure prediction**. *BMC Bioinformatics* 2008, **9**(40).
3. Roy A, Kucukural A, Zhang Y: **I-TASSER: a unified platform for automated protein structure and function prediction**. *Nature Protocols* 2010, **5**(4).
4. Wu S, Zhang Y: **A local meta-threading server for protein structure prediction**. *Nucleic Acids Res* 2007, **35**:3375-3382.
